# Phenotypic characterization of two neuroinvasive Toscana virus strains clinically associated with self-limited and persistent infections in human neural cells and brain organoids

**DOI:** 10.3389/fcimb.2025.1658107

**Published:** 2025-09-25

**Authors:** Stefania Vogiatzis, Gianni Gori Savellini, Chiara Terrosi, Marta Trevisan, Gabriele Anichini, Letizia Rizzo, Giulia Alessandri, Emanuela Dal Molin, Camilla Lucca, Luisa Barzon, Maria Grazia Cusi

**Affiliations:** ^1^ Department of Molecular Medicine, University of Padova, Padova, Italy; ^2^ Virology Unit, Department of Medical Biotechnologies, University of Siena, Siena, Italy; ^3^ Microbiology and Virology Unit, Padova University Hospital, Padova, Italy; ^4^ Microbiology and Virology Unit, ‘Santa Maria delle Scotte’ University Hospital, Siena, Italy

**Keywords:** Toscana virus, neurotropism, brain organoids, neuronal cells, β-interferon, viral persistence

## Abstract

**Background:**

Toscana virus (TOSV), a sandfly-borne phlebovirus, is a frequent cause of viral meningitis and meningoencephalitis in Mediterranean countries during summer months. Clinical outcomes vary from self-limited disease to prolonged persistent infection lasting over 3 months, but the mechanisms underlying these differences remain unclear. In this study, we compared the pathobiological features of two clinical TOSV strains, SI-1812 (associated with self-limited disease) and INMI (associated with persistent infection), using human neural models.

**Methods:**

Three human neural systems, DBTRG.05MG glioblastoma cells, embryonic stem cell-derived neurons, and human brain organoids (hBOs), were infected with the two TOSV strains. Viral replication, cytopathic effect, innate immune response, and viral protein expression were assessed. Furthermore, whole-genome sequencing was performed to identify strain-specific differences.

**Results:**

The INMI strain showed reduced replication and cytopathic effect compared to SI-1812, supporting persistent infection in hBOs for up to 21 days. SI-1812 infection triggered a strong interferon-β response even at early stages of infection and low viral titers, whereas INMI induced a modest innate immune response in the early stages of infection, likely supporting its persistence in hBOs. The timing of viral NSs protein expression differred between the two viral strains suggesting distinct mechanisms in RIG-I activation and inflammatory response modulation.

**Conclusions:**

Distinct host-pathogen interactions underlie the divergent clinical courses of TOSV strains. The findings provide insights into mechanisms of viral persistence in the human brain and may guide future therapeutic strategies.

## Introduction

Toscana virus (TOSV) is a vector-borne virus, first isolated from *Phlebotomus perniciosus* and *Phlebotomus perfiliewi* in central Italy in 1971. This virus belongs to the *Phlebovirus* genus of the *Phenuiviridae* family, order *Hareavirales* (Verani et al., 1982; Charrel et al., 2012). It causes a variety of clinical syndromes ranging from a brief self-limiting febrile illness to retinitis, encephalitis, or meningoencephalitis. The viral particle has an envelope with two glycoproteins (G_N_ and G_C_) and a tripartite (L, M, and S) single-stranded RNA genome of negative polarity (Walter and Barr, 2011; Olschewski et al., 2020). TOSV, like other phleboviruses, uses envelope proteins for entry into target cells through interaction with host receptors and for the assembly of progeny particles in infected cells (Spiegel et al., 2016; Koch et al., 2023). Entry receptors and cofactors for TOSV in host cells are only partially known. TOSV subverts heparan sulfates (HS) glycosaminoglycan and C-type lectins, DC-SIGN and L-SIGN, to attach to the cell surface (Lozach et al., 2011; de Boer et al., 2012; Hofmann et al., 2013; Pietrantoni et al., 2015; Riblett et al., 2015; Léger et al., 2016; Tani et al., 2016). In the dermis, TOSV infects dendritic cells (DCs) through interaction with the C-type lectin DC-SIGN, which functions as an endocytic receptor (Lozach et al., 2011; Hofmann et al., 2013; Léger et al., 2016), while other C-type lectins (L-SIGN and LSECtin) may act as receptors on the surface of endothelial cells (Léger et al., 2016; Tani et al., 2016). The mechanism of TOSV invasion of the central nervous system (CNS) and the host receptors and factors involved in neuroinvasion remain unknown. In humans, symptoms of TOSV infection occur after an incubation period of 3–7 days and typically persist for about 7–12 days (Charrel et al., 2012; Ayhan and Charrel, 2020). Most TOSV infections in humans are asymptomatic or pauci-symptomatic, presenting with flu-like symptoms such as headache, fever, nausea, or vomiting (Ayhan and Charrel, 2020). A high rate of asymptomatic or mild TOSV infections is suggested by studies in endemic areas reporting seroprevalences ranging from 5% to 30% (Valassina et al., 2003; Sanbonmatsu-Gámez et al., 2005; Calamusa et al., 2012; Cusi et al., 2013; Marchi et al., 2017; Gori Savellini et al., 2020). Patients with CNS involvement may present with aseptic acute meningitis, encephalitis, or meningoencephalitis (Varani et al., 2015; Ayhan and Charrel, 2020). The clinical picture of meningitis caused by TOSV is similar to that caused by other viral agents. However, levels of anti-inflammatory and antiviral mediators are significantly higher in the cerebrospinal fluid (CSF) of TOSV-infected patients than in those with other infectious or noninfectious neurological diseases (Verani et al., 1991; Braito et al., 1998; Hemmersbach-Miller et al., 2004; Varani et al., 2015). Other clinical manifestations are associated with peripheral neuropathy, including paraesthesia, hyporeflexia, hypotonia, imbalance, hyperaesthesia, and Guillain–Barré syndrome (Sanbonmatsu-Gámez et al., 2009; Zanelli et al., 2013; Gori Savellini et al., 2015; Okar et al., 2021; Matusali et al., 2022). Rare atypical presentations, such as epididymo-orchitis during the acute phase, have been reported, with TOSV (INMI strain) isolated from the seminal fluid of a patient with meningoencephalitis and persistent TOSV shedding in semen up to 59 days postsymptom onset (Zanelli et al., 2013; Matusali et al., 2022). Despite the clinical and epidemiological relevance of TOSV, strain-specific phenotypic differences—particularly those linked to persistent infections or diverse clinical outcomes—remain poorly characterized. Understanding these differences is crucial for improving patient management, guiding therapeutic strategies, and informing public health interventions. Characterizing strains such as TOSV INMI, which is associated with persistent infection, could provide novel insights into viral persistence mechanisms and host immune interactions that shape clinical outcomes. It is already known that the nonstructural NS protein of TOSV and other phleboviruses functions as an innate immune suppressor, delaying the host’s response to infection. This viral protein, which acts as an E3 ubiquitin ligase to inhibit type I interferon beta (IFN-β) expression, may play a critical role in determining viral phenotype (Gori-Savellini et al., 2013; Gori Savellini et al., 2015; Gori Savellini et al., 2019). This study aims to phenotypically characterize the TOSV INMI strain in comparison with a reference strain (SI-1812) (Baldelli et al., 2004), using multiple neural cell models, including the human glioblastoma cell line DBTRG.05MG, human cortical neurons, and brain organoids derived from embryonic stem cells, to investigate infection and replication dynamics. Such detailed analysis will contribute to a deeper understanding of how strain-specific differences influence TOSV pathogenesis and persistence.

## Material and methods

African green monkey’s epithelial kidney cells, Vero E6 (ATCC CRL-1586), were cultured in Dulbecco’s modified Eagle’s medium (DMEM) (EuroClone, Milan, Italy) supplemented with 100 U/ml penicillin/streptomycin (EuroClone) and 5% heat-inactivated fetal bovine serum (FBS) (EuroClone) at 37°C in a 5% CO_2_-supplemented incubator. TOSV strains SI-1812 and INMI were respectively isolated from the CSF and from the seminal fluid of patients with meningitis on Vero E6 cells at the Virology Laboratory of S. Maria delle Scotte Hospital in Siena, Italy. After isolation and characterization, viruses were propagated on Vero E6 cells, and stocks were prepared and stored at − 80 °C. DBTRG.05MG human glioblastoma cells (ATCC CRL-2020) were cultured in RPMI medium (EuroClone) supplemented with 100 U/ml penicillin/streptomycin and 10% FBS at 37 °C in a 5% CO_2_-supplemented incubator.

### Generation of neurons from human ESCs

To obtain human neurons, AAVS1-TRE3G-NGN2 human embryonic stem cells (hESC-NGN), kindly provided by J.W. Harper, were used and differentiated as indicated by Ordureau et al., with minor modifications (Ordureau et al., 2018). Briefly, H9-NGN2 cells were seeded on day 0 in a Geltrex^®^-coated six-well plate (2 × 10^5^ cells/well) in ND1 medium composed of DMEM/F12/NEAA (Invitrogen, Thermo Fisher Scientific, Waltham, MA, USA), human Brain-Derived Neurotrophic Factor (BDNF) (10 ng/ml; PeproTech, Thermo Fisher Scientific), human NT-3 (10 ng/ml, PeproTech, Thermo Fisher Scientific), human laminin (0.2 µg/ml, Invitrogen, Thermo Fisher Scientific), and doxycycline (2 µg/ml, Clontech, Takara Bio USA, San Jose, CA, USA). On day 1, ND1 medium was replaced with fresh ND1 medium; on day 2, ND1 medium was replaced with ND2 medium consisting of Neurobasal medium supplemented with B27/GlutaMAX (Invitrogen, Thermo Fisher Scientific), BDNF, NT3, and doxycycline (concentrations are the same as above). After day 2, 50% of the medium in each well was replaced every 2 days with fresh medium. On day 7, cells were detached by treatment with Accutase (Gibco, Thermo Fisher Scientific) and replated in Geltrex^®^-coated six-well plates (4 × 10^5^ cells/well) in ND2 medium supplemented with Y27632 (10 µM, Thermo Fisher Scientific). On day 8, the medium was replaced with fresh ND2 Medium. After day 8, 50% of the medium in each well was replaced every 2 days until day 14, when cortical neurons reached the mature phase.

### Generation of human brain organoids from human ESCs

To obtain 3D human brain organoids (hBOs), the protocol developed by Lancaster and Knoblich was adopted with some modifications (Lancaster and Knoblich, 2014). Briefly, on day 0, human ES cells (H9, WiCell Institute, Madison, U.S.A.) were dissociated to a single-cell suspension and seeded in round-bottom, low-attachment 96-well plates (1 × 10^5^ cells/well) in mTeSR complete medium (StemCell Technologies, Cologne, Germany) supplemented with Y27632 (10 µM, Thermo Fisher Scientific). On day 3, the medium was replaced with fresh mTeSR. On day 5, the medium was replaced with Neural Induction Medium, composed of DMEM/F12/NEAA (Gibco, Thermo Fisher Scientific) with GlutaMAX/B27 (Thermo Fisher Scientific), heparin (1 mg/ml, Merck Millipore, Burlington, MA, USA), and antibiotic–antimycotic (Anti-Anti, 1% v/v, Gibco, Thermo Fisher Scientific). The embryoid bodies (EBs) were fed every other day. On day 11, the EBs were embedded on Matrigel (Corning™, Merck Millipore) droplets and grown in a low-attachment six-well plate (eight to nine EBs/well) in differentiation medium (DM), composed of Neurobasal/DMEM/F12 (Thermo Fisher Scientific) supplemented with GlutaMAX/NEAA/B27/N2 (Thermo Fisher Scientific), with human insulin (0.00025%, v/v, Merck-Milliore, Milan, Italy) and 2-mercaptoethanol (0.00035%, v/v, Gibco, Thermo Fisher Scientific) added. To prevent contamination, Anti-Anti (1%, v/v, Gibco, Thermo Fisher Scientific) was included. On day 13, DM was replaced with fresh medium. On day 15, the medium in each well was replaced with differentiation medium 2 (DM2), i.e., DM with B27 plus vitamin A (Thermo Fisher Scientific), and the plate was placed on an orbital shaker. From days 15 to 60, the hBOs were grown in an orbital shaker until the day of infection. The medium was changed twice per week.

### Viral infection and analysis of viral replication kinetics in neural cell cultures and organoids

DBTRG.05MG cells and human ESC-derived neurons were infected with SI-1812 or INMI TOSV strains at an MOI of 0.1. After 1 h of virus absorption, extensive washes were performed to remove the inoculum, and complete growth medium was added. Cell culture supernatants were collected at 1, 3, 5, 6, and 7 days postinfection (p.i.) and stored at − 80 °C for further analysis. hBOs were infected with SI-1812 and INMI TOSV strains at an MOI of 0.2. For infection, hBOs were transferred into a tube containing the required amount of virus. After inoculation, hBOs were centrifuged at 1,100 rpm for 30 min. Subsequently, hBOs were incubated at 37 °C with 5% CO_2_ for 60 min to allow the adsorption of the virus. The medium was then removed, the organoids were washed with PBS twice, and fresh DM2 was added. The organoids were incubated at 37 °C, with 5% CO_2_ in an orbital shaker. Supernatants of infected organoids were collected at different time points (1, 3, 6, 10, 16, 19, and 21 days postinfection) for further analysis. The organoids were also collected to perform immunofluorescence. The release of infectious viral particles by infected DBTRG.05MG cells, human ESC-derived neurons, and hBOs was determined by virus microtitration assay on Vero E6 cells cultured in 96-well plates, and the 50% Tissue Culture Infectious Dose (TCID_50_/ml) end point titer was calculated using the Reed and Muench method (Reed and Muench, 1938). Results were reported as mean values ± standard deviation (SD) from at least three independent experiments.

### Assessment of IFN-β production

Medium from DBTRG.05MG cells and human ESC-derived neurons, mock- or TOSV-infected, collected at 1, 3, 5, 6, and 7 days p.i as described above, and the medium from hBOs, mock- or TOSV-infected, collected at 1, 3, 6, 10, 16, 19, and 21 days p.i., were processed for IFN*-*β quantification by enzyme-linked immunosorbent assay (ELISA) using a commercial kit (VeriKine-HS Human IFN Beta TCM ELISA Kit; PBL Assay Science, Piscataway, NJ, USA), following manufacturer’s instructions. IFN-β level was expressed as mean cytokine concentrations (pg/ml) ± SD from at least three independent experiments.

### Multiplex cytokine detection

Cell supernatant of mock-infected or TOSV-infected hBOs was centrifuged at 10,000 × *g* for 5 min, prior to performing the assay, and diluted 1:4 in Universal Assay Buffer. A multiplex biometric ELISA-based immunoassay, containing dyed microspheres conjugated with 10 different monoclonal antibodies specific for a target cytokine, was used according to the manufacturer’s instructions (Human Luminex Discovery Assay, Bio-Techne, Minneapolis, MN, USA). Magnetic bead concentrations were measured using a Luminex™ detection system (Luminex™ 200™ Instrument System, Bio-Techne, Minneapolis, MN, USA). The following cytokines were analyzed: IFN-β, Interferon gamma (IFN-γ), tumor necrosis factor (TNF-α), interleukin (IL)-1β, IL-2, IL-4, IL-6, IL-8, IL-10, and IL-12. The analyte concentration was calculated using a 7-point standard curve. Acquired data were analyzed using xPONENT v4.3.309.1, and cytokine concentrations were reported as picograms per milliliter.

### Immunofluorescence analysis

Neurons (10^4^ cells/cover slip) were grown on Geltrex^®^-treated coverslips. Cells were fixed in paraformaldehyde (4%, v/v, Thermo Fisher Scientific) for 20 min and permeabilized with Triton X-100 (0.1%, v/v) in Dulbecco's modified Phosphate Buffered Solution (DPBS) (Gibco, Thermo Fisher Scientific) for 5 min at room temperature. Cells were then incubated with the following primary antibodies diluted 1:100 in BSA (1%, w/v, in PBS): serum anti-TOSV, beta-3 Tubulin (Thermo Fisher Scientific). Nuclei were stained with the DRAQ5™ intercalating fluorescent probe (Thermo Fisher Scientific), and cells were observed with a confocal microscope (Leica, Buffalo Grove, IL, USA) at × 63 magnification and processed using Fiji-ImageJ software. Human brain organoids were fixed in paraformaldehyde (4%, v/v, Thermo Fisher Scientific) for 40 min and washed three times in DPBS. hBOs were placed in 10% w/v sucrose solution for 30 min, in 20% w/v sucrose solution for 30 min, and finally in 30% w/v sucrose solution at + 4 °C, until embedding with OCT solution (VWR International PBI, Milan, Italy) in a plastic mold. To solidify the solution rapidly, molds were put in contact with liquid nitrogen vapors. These organoids were prepared for cryosectioning and immunostaining. The 20-µm-thick sections generated were washed three times with DPBS and incubated with 4% BSA/DPBS containing 0.2% Triton X-100 for 1 h at room temperature. After washing twice in DPBS, primary antibodies diluted in 4% BSA/PBS were added and incubated overnight at 4 °C. Cells were incubated with the following primary antibodies: serum anti-TOSV, anti-TUJI1 (Thermo Fisher Scientific), and anti-cleaved caspase-3 (Asp175; Cell Signaling Technology, Leiden, The Netherlands). Nuclei were stained with DRAQ5™ interlayer fluorescent probe (Thermo Fisher Scientific), and cells were observed confocal microscope (Leica, Buffalo Grove, IL, USA) and processed by Fiji-ImageJ software. The experiment was repeated three times, and a total of 12 slices of processed brain tissue were analyzed. Vero E6 cells were infected with medium collected from hBOs at 6 days p.i. and fixed with cold acetone:methanol solution for 10 min. Immunofluorescence staining was then performed using an anti-N TOSV antibody, followed by a FITC-conjugated secondary antibody. Images were acquired with a fluorescence-equipped Eclipse Ts2 microscope (Nikon, Milan, Italy) and processed using Fiji-ImageJ software. Corrected total cell fluorescence (CTCF) was calculated as: CTCF = Integrated Density − (Area of selected cell × Mean fluorescence of background readings). Data are presented as mean CTCF ± SD from at least three independent fields.

### Western blot analysis

Endogenous Retinoic-acid Inducible Gene I (RIG-I) and TOSV SI-1812 or INMI NS expression was investigated in DBTRG.05MG cells infected as previously described and collected at 24, 48, and 72 h p.i. Whole-cell lysates were prepared in RIPA buffer (25 mM Tris-HCl [pH 7.5]; 150 mM NaCl; 1% Triton X-100) supplemented with COmplete™ Protease Inhibitor (Merck-Millipore), and protein concentration was determined by BCA assay (Pierce, Milan, Italy). Fifty micrograms of total proteins, supplemented with Laemmli sample buffer and denatured for 5 min at 95 °C, was separated by SDS-PAGE and then transferred to a NitroBind nitrocellulose membrane (Santa Cruz Biotechnology, Heidelberg, Germany). After blocking with 5% nonfat dry milk, the filters were incubated O/N at room temperature with anti-RIG-I CARDs (1:5,000), anti-NSs (1:200), or antiactin (1:2,500; Invitrogen, Thermo Fisher Scientific) as a loading control. After being washed three times with PBS containing 0.05% Tween-20 (PBS-T), membranes were incubated with a horseradish peroxidase (HRP)-conjugated anti-mouse IgG secondary antibody (Merck-Millipore). After several washes, the target bands were visualized using an enhanced chemiluminescence kit (Pierce), and images were acquired with a ChemiDoc instrument (BioRad, Milan, Italy).

### Statistical analysis

Statistical significance was assessed with the two-tailed Chi-square test, paired or unpaired as appropriate. Results were considered statistically significant at *p* < 0.05. When appropriate, values are reported as mean ± SD. One-way ANOVA followed by a Bonferroni *post hoc* test was performed to compare the significance among different study groups. Data are presented as mean values ± SD of three independent experiments performed in triplicate, unless otherwise stated. All analyses were performed using GraphPad Prism software (v.8.0.1).

## Results

### TOSV growth kinetics in DBTRG.05MG human glioblastoma cells

**Figure 1:**
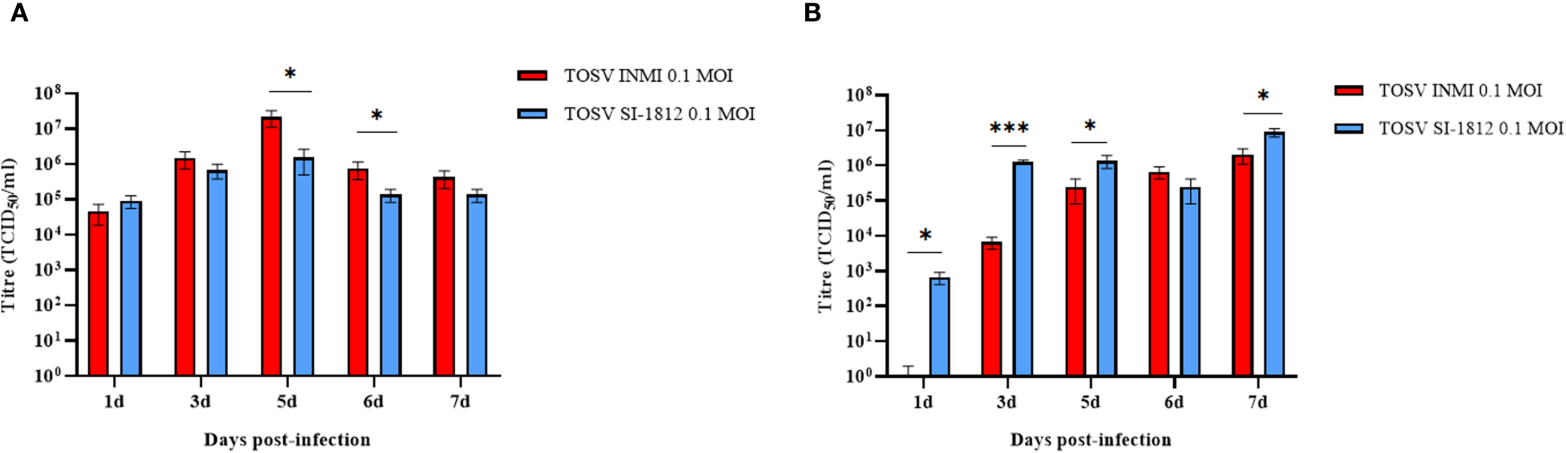
TOSV SI-1812 and INMI strains’ kinetic growth in DBTRG.05MG glioblastoma cell line and ESC-derived neurons. Representation of viral titer in **(A)** DBTRG.05MG glial cells or **(B)** ESC-derived neurons infected with 0.1 MOI of TOSV INMI and SI-1812 strains at different time points p.i. (1, 3, 5, 6, and 7 days). Data are reported as mean TCID_50_/ml ± SD from at least three independent experiments. *p*-values, derived from ANOVA multicomparison tests (Dunnett), are reported as ^*^ *p* < 0.05; ^**^ *p* < 0.01; ^***^ *p* < 0.001.

To compare the infection and replication efficiency of the two TOSV strains (INMI and SI-1812) in CNS cells, we used different human cell systems, i.e., the DBTRG.05 MG glioblastoma cell line, cortical neurons, and brain organoids. The DBTRG.05 MG cell line, isolated from a female patient with glioblastoma, although highly aneuploid, responds to IFN-β stimulus with expression of proinflammatory genes, thus suggesting that it originated from astrocytes. Both TOSV INMI and SI-1812 strains were able to infect and grow in DBTRG.05 MG glioblastoma cells, although the INMI strain reached higher titers at 5 days (2.1 × 10^7^ TCID_50_/ml ± 1.05 × 10^7^ TCID_50_/ml, *p* = 0.03) and 6 days p.i. (7.5 × 10^5^ TCID_50_/ml ± 3.8 × 10^5^ TCID_50_/ml, *p* = 0.04) than the SI-1812 strain (1.5 × 10^6^ ± 1.06 × 10^6^ and 1.4 × 10^5^ ± 5.5 × 10^4^ TCID_50_/ml, respectively). At 7 days p.i., viral titer slightly decreased for both the strains (**Figure 1A**).

### TOSV growth kinetics in human ESC-derived neurons

**Figure 2:**
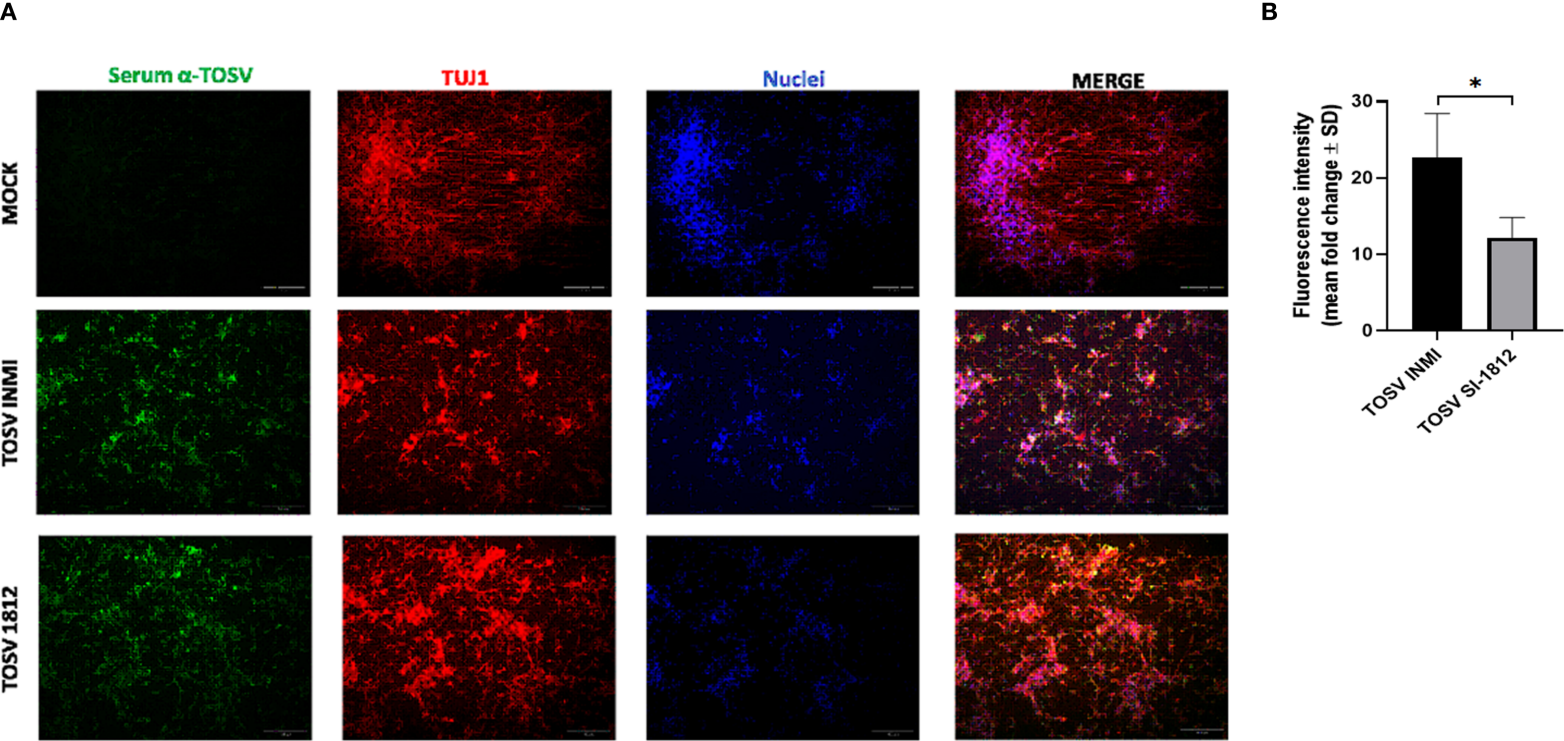
Immunofluorescence of TOSV-infected human neurons. INMI and SI-1812 TOSV infection of human neurons was evaluated by immunostaining with anti-TOSV serum (green), anti-TUJ1 antibody (red), and DAPI (blue, nuclei) at 7 days p.i. Fluorescence intensities were quantified from four images per condition using ImageJ software. Data are presented as means ± SD from three independent experiments. Statistical significance was assessed using a two-tailed unpaired Student’s *t*-test, with ^*^ *p* < 0.05 considered significant. Scale bar, 100 µm.

Differentiated cortical neurons were infected with TOSV INMI and SI-1812 at an MOI of 0.1 and assayed during the next 7 days. These cells were susceptible to TOSV infection and permissive to replication, as determined by titration of infectious viral particles and immunofluorescence. As shown in **Figure 1B**, in this neural cell system, TOSV INMI replication was slower than that of the SI-1812 strain in the first 3 days p.i., yielding significantly lower infectious viral titer in cell supernatant (day 3 p.i., SI-1812 titer 1.2 × 10^6^ TCID_50_/ml ± 1.7 × 10^5^ TCID_50_/ml vs. 6.6 × 10^3^ TCID_50_/ml ± 2.5 × 10^3^ TCID_50_/ml of the INMI strain, *p* = 0.028). However, the INMI strain appeared more capable of spreading in these cells than the SI-1812 strain. Indeed, although both strains reached a similar viral titer at 6 days p.i. (*p* = 0.074), immunofluorescence showed a higher number of positive cells in neurons infected with the INMI strain (*p* = 0.028) (**Figure 2A**). Furthermore, the fold-change fluorescence relative to the mock sample confirmed the enhanced capability of the INMI strain to spread in ESC-derived neurons (**Figure 2B**).

### TOSV growth kinetics in human brain organoids

**Figure 3:**
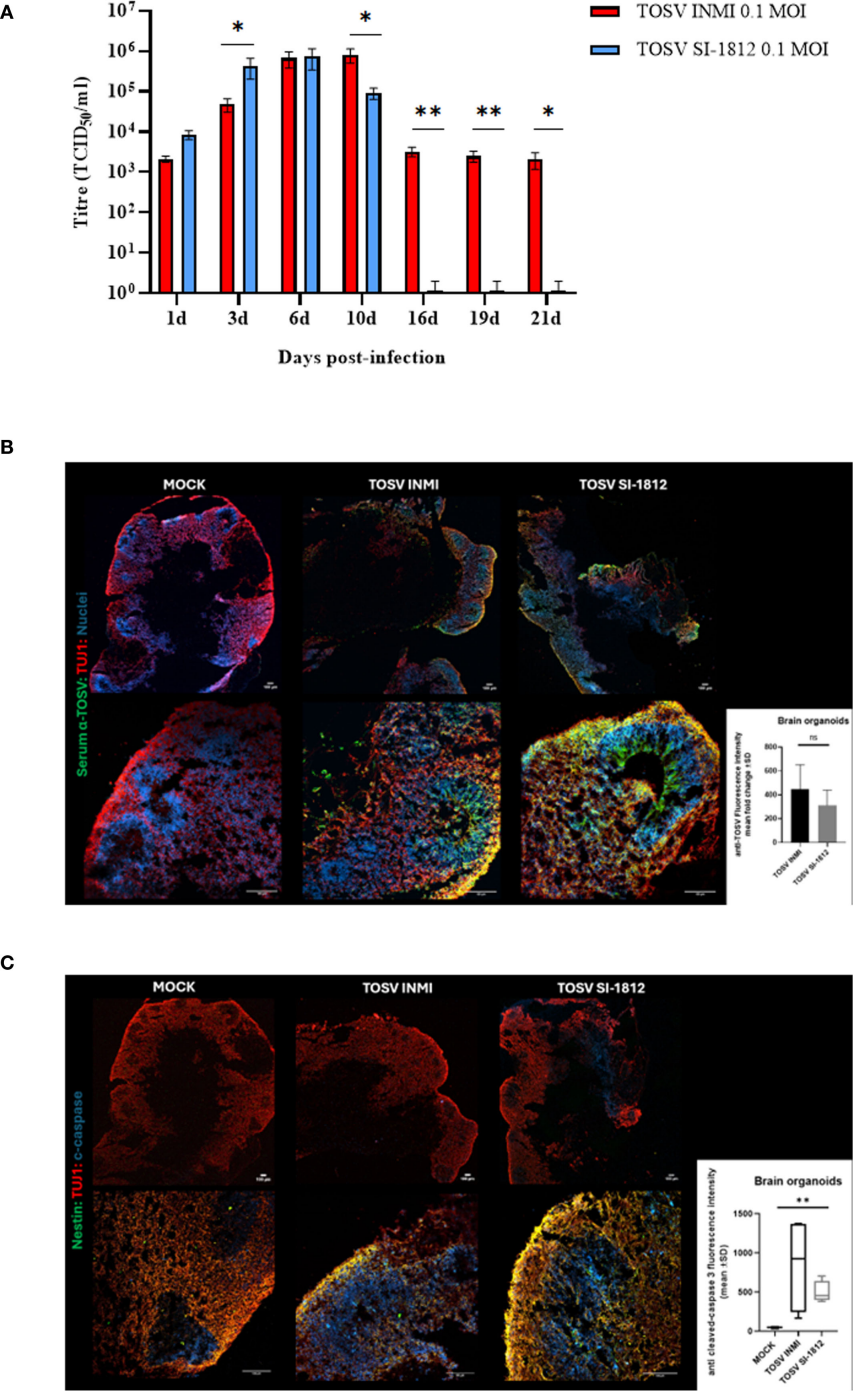
TOSV infection, replication, and apoptosis induction in human brain organoids. **(A)** Representation of viral titer (TCID_50_/ml) in human brain organoids infected with 0.2 MOI of TOSV INMI and SI-1812 strains at different time points after infection (1, 3, 6, 10, 16, 19, and 21 days). *p*-values for differences between the two strains are reported as ^*^ *p* < 0.05, ^**^ *p* < 0.01, and ^***^ *p* < 0.001. **(B)** TOSV infection at 0.2 MOI in human brain organoids (hBO) was evaluated by immunostaining with anti-TOSV serum (green), anti-TUJ1 (red), and Draq5 (blue, nuclei) at 6 days postinfection (magnifications: × 4 and × 20). Images are representative of three independent experiments. The bar graph represents anti-TOSV fluorescent intensity in human brain organoids as the mean gray value. Data are presented as mean ± SD from *n* = 3 independent experiments per group. *p*-values were derived from an unpaired *t*-test. ns, not statistically significant. **(C)** Evaluation of TOSV infection in human brain organoids (hBO) by immunostaining with Nestin (green), anti-TUJ1 (red), and cleaved caspase-3 (blue) at 6 days postinfection with 0.2MOI TOSV (magnifications: × 4 and × 20). Images are representative of three independent experiments. The bar graph shows the mean gray value ± SD of cleaved caspase-3 fluorescence intensity in human brain organoids from *n* = 4 images for each group. *p*-values were derived from the nonparametric Kruskal–Wallis test (^**^ *p* < 0.001).

**Figure 4:**
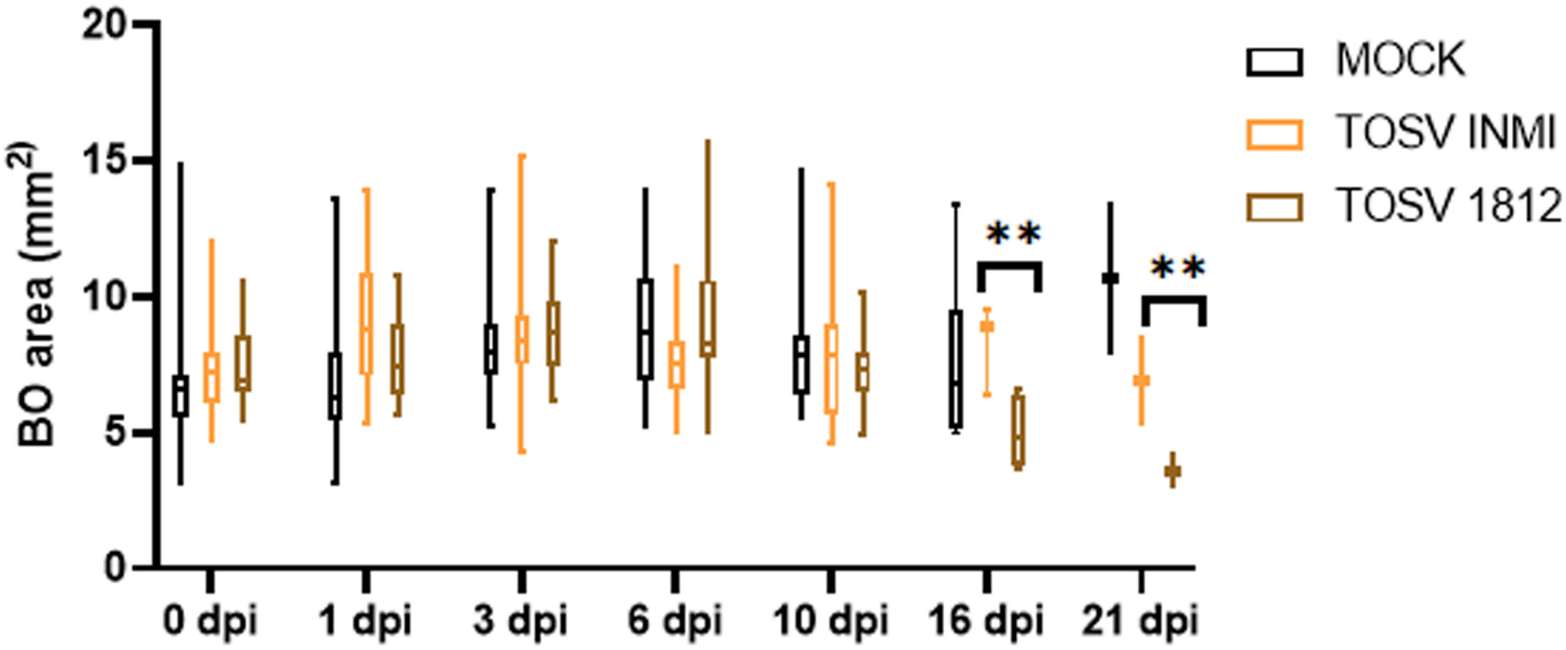
Effect of TOSV infection on brain organoids. Brain organoid size was measured in mock-infected (black), TOSV INMI-infected (orange), and TOSV SI-1812-infected (brown) groups. Organoid area was measured on different days p.i. (0, 1, 3, 6, 10, 16, and 21). Data are represented as whisker plots showing mean ± SEM from *n* = 5 replicates per group. The *p*-values were derived by ANOVA multicomparisons test (Dunnett) between mock and TOSV-infected strains ^(**^ *p* < 0.001).

hBOs were infected at day 60 of differentiation, when they presented complex cell–cell and cell–environment interactions similar to the *in vivo* condition. Briefly, hBOs were infected with TOSV INMI and SI-1812 strains at a 0.2 MOI. Experiments were conducted in biological triplicate, with five hBOs per experiment. Following infection, morphological sizing of hBOs was monitored up to day 21 p.i. Viral infection kinetics and replication efficiency were assessed by infectious virus titration for each experiment. Additionally, immunofluorescence staining was performed on hBOs, with a total of six slides per organoid (**Figure 3**). Also in this cell system, the TOSV INMI strain grew more slowly than the SI-1812 strain for the first 3 days p.i. (*p* = 0.045). At day 6 p.i., the viral titer (about 10^5^ TCID_50_/ml) was similar for the two strains (*p* = 0.808). At 10 days p.i., however, the INMI strain showed a higher titer (*p* = 0.017) (8.1 × 10^5^ TCID_50_/ml ± 3.1 × 10^5^ TCID_50_/ml) than the SI-1812 strain (9.1 × 10^4^ TCID_50_/ml ± 2.7 × 10^4^ TCID_50_/ml) (**Figure 3A**). The difference further increased in the follow-up due to massive cell death and hBO disruption. Indeed, as shown in **Figure 4**, the size of SI-1812-infected hBOs was smaller than that of hBOs infected with INMI at day 16 p.i. (*p* < 0.0009). Immunofluorescence performed on TOSV-infected hBOs at day 6 p.i. showed a similar distribution of infected cells for both strains (**Figure 3B**), but a higher level of apoptosis induction in INMI-infected hBOs compared to SI-1812-infected hBOs, as shown by coimmunostaining for cleaved caspase-3 (*p* < 0.0008, **Figure 3C**).

### Replication efficiency and cytopathic effect of INMI and SI-1812 TOSV strains in Vero cells

**Figure 5:**
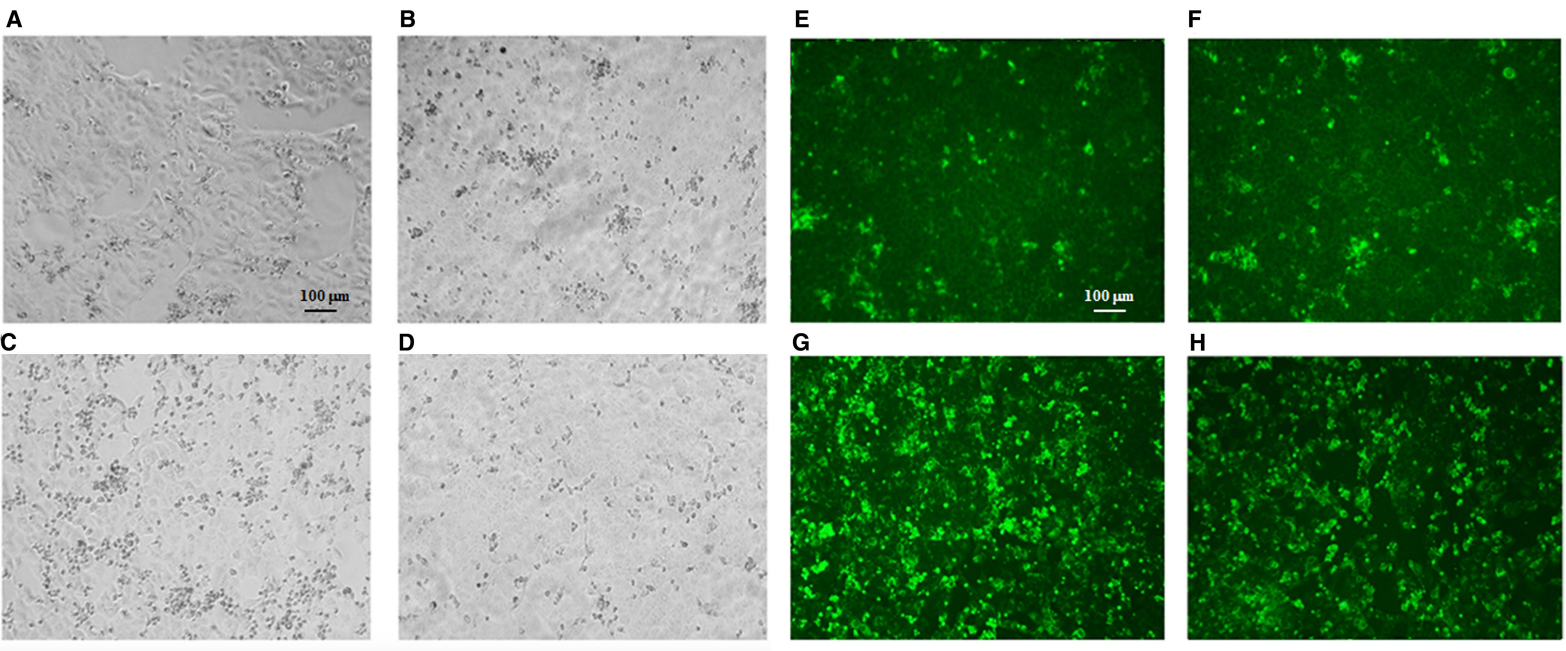
TOSV viability from infected human brain organoids. Representative cytopathic effect (CPE) of TOSV strains isolated from brain organoids at 6 days p.i. on Vero E6 cells. At 3 days p.i. **(A)** TOSV INMI strain and **(B)** TOSV SI-1812 strain; at 4 days p.i. **(C)** TOSV INMI strain and **(D)** TOSV SI-1812 strain. Immunofluorescence with anti-TOSV mice serum of the same cells stained at 3 days p.i. **(E)** TOSV INMI and **(F)** TOSV SI-1812 strains; and at 4 days p.i. **(G)** TOSV INMI and **(H)** TOSV SI-1812 strains (magnification: × 10).

The two virus strains isolated from brain organoids at 6 days p.i., showing similar titer, were then inoculated in Vero E6 cells. At 3 days p.i., SI-1812 TOSV showed a CPE that was slightly more pronounced than the other virus. In contrast, at 4 days p.i., the CPE was markedly more extended in cells infected with the INMI strain than those infected with SI-1812, suggesting a greater spreading capability for this strain (**Figures 5A–D**). This result was further confirmed by immunofluorescence, which demonstrated the presence of the virus in a larger number of cells infected with the INMI strain at 4 days p.i. (**Figures 5E–H**), which, in turn, exhibited a reduced cytopathic effect and a decreased neural cell death. Indeed, the mean CTCF for infected INMI cells was 7.45 × 10^7^ ± 1.87 × 10^7^, while for SI-1812 samples, it was 2.56 × 10^7^ ± 1.27 × 10^7^ (*p* = 0.002).

### Interferon-β induction in TOSV-infected cells

**Figure 6:**
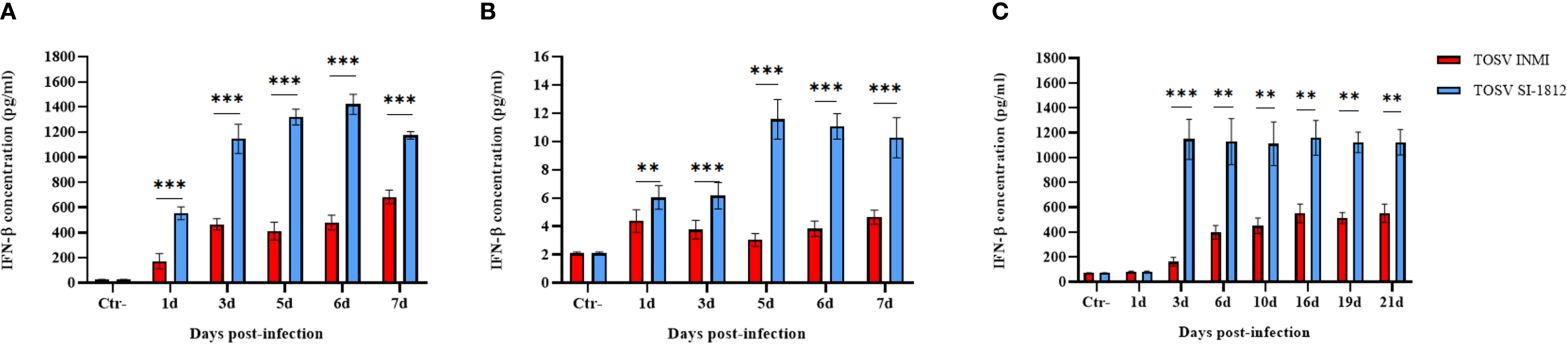
Induction of IFN-β in neural cells infected with TOSV. Representation of INF-β production in **(A)** DBTRG.05MG glial cells, **(B)** human ESC-derived neurons infected with 0.1 MOI of TOSV INMI and SI-1812, and **(C)** human brain organoids (hBOs) infected with 0.2 MOI of the same virus strains at different time points p.i. (1, 3, 5, 6, and 7 days). Negative controls (Ctr^−^) are represented by noninfected glial cells. Graphs represent mean cytokine concentration (pg/ml) ± SD from at least three independent experiments. The *p*-value of the differences between the two strains was derived by ANOVA multicomparisons test (Dunnett) and reported as ^*^ *p* < 0.05, ^**^ *p* < 0.01, and ^***^ *p* < 0.001.

Interferon-β expression was evaluated in human DBTRG.05MG glioblastoma cells, ESC-derived human cortical neurons, and hBOs infected with the two strains of TOSV at different time points. Infection with TOSV INMI led to a significantly lower induction of IFN-β than the SI-1812 strain in the two cell types and hBOs (**Figure 6**). In infected DBTRG.05MG cells, the highest level of IFN-β was recorded at day 6 p.i. (478.46 pg/ml ± 59.51 pg/ml of the INMI strain vs. 1,427.57 pg/ml ± 80.19 pg/ml of the SI-1812 strain; *p* < 0.0001). It then began to drop only in cells infected with SI-1812, conceivably as a consequence of cell death due to cytopathic effect (**Figure 6A**). Similarly, along the 7-day time course in ESC-derived neurons, IFN-β was produced at lower levels in cells infected with the INMI strain (mean concentration: 3.6 pg/ml ± 0.93 pg/ml) than in those infected with the SI-1812 strain (mean concentration: 7.8 pg/ml ± 3.7 pg/ml), which showed a significant and progressive increase in IFN-β secretion, doubling its level after the third day p.i. (6.1 pg/ml ± 0.92 pg/ml) until the seventh day p.i. (10.2 pg/ml ± 1.41 pg/ml) (*p* < 0.0001) (**Figure 6B**).

**Figure 7:**
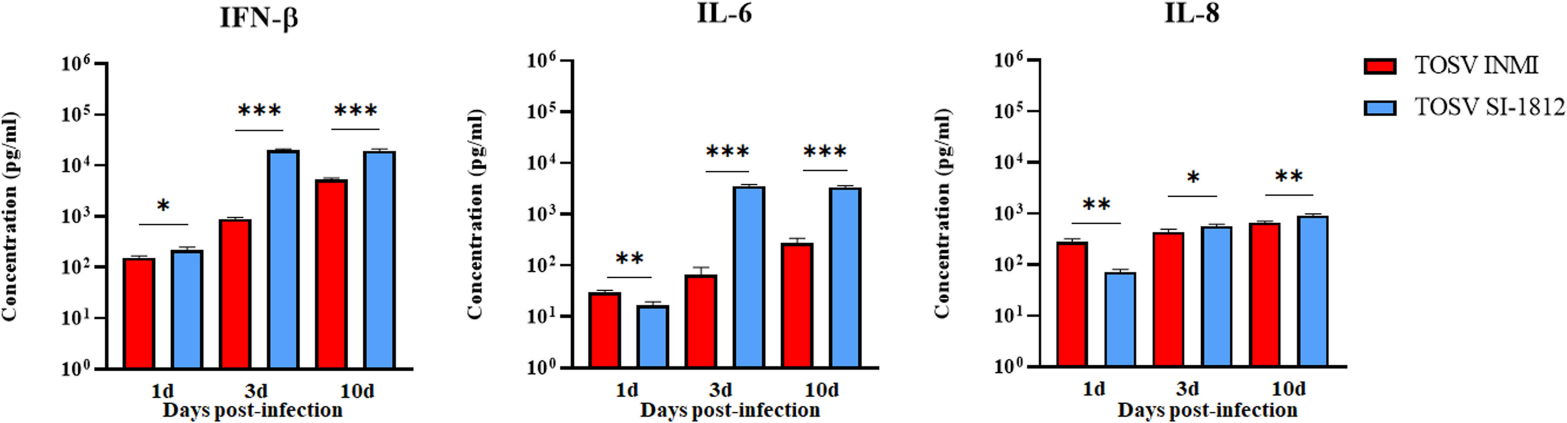
Analysis of pro- and anti-inflammatory cytokines in TOSV-infected human brain organoids. Representation of IFN-β, IL-6, and IL-8 concentrations in supernatants of hBOs infected with TOSV INMI and SI-1812 strains and collected at 1, 3, and 10 days p.i. Uninfected organoids served as negative controls (Ctr^−^). Among the analyzed cytokines, only IFN-β, IL-6, and IL-8 showed significant modulation due to viral infection. Data are presented as mean concentrations (pg/ml) ± SD from at least three independent experiments. Statistical analysis was performed using a two-tailed one-way ANOVA test (Dunnett) and reported as ^*^ *p* < 0.05, ^**^ *p* < 0.01, and ^***^ *p* < 0.001.

In hBOs, the difference of IFN-β induction by the two strains was more pronounced, with IFN-β levels about 10-fold lower in cells infected with the TOSV INMI strain (161 pg/ml ± 35.4 pg/ml) than in cells infected with the SI-1812 strain (1,148 pg/ml ± 161.3 pg/ml) at 3 days p.i. (*p* = 0.0005) (**Figure 6C**). In cells infected with the INMI strain, IFN-β increased at 6 days p.i. (398 pg/ml ± 55.4 pg/ml) and showed no significant differences in the following days until day 21 p.i. (552 pg/ml ± 74.23 pg/ml) (**Figure 6C**). Multiplex cytokine analyses carried out on the growth medium of infected hBOs confirmed the higher production of IFN-β (day 10 p.i.: 1.9 × 10^4^ pg/ml ± 1.5 × 10^3^ pg/ml, *p* = 0.0001), as well as the proinflammatory cytokine IL-6 (day 10 p.i.: 3.4 × 10^3^ pg/ml ± 2.1 × 10^2^ pg/ml, *p* < 0.0001) and IL-8 (day 10 p.i.: 922 pg/ml ± 57.2 pg/ml, *p* = 0.0035) in cells infected with SI-1812, compared to those infected with INMI (**Figure 7**). The other assayed interleukins were not detected. The high expression of IL-6 and IL-8 in the supernatant of SI-1812-infected cells demonstrated that this strain unveiled the neuropathogenesis of the brain by inducing a strong inflammation and destroying cells, in contrast to the INMI strain, which persisted longer in hBOs.

### RIG-I expression in DBTRG.05MG-infected cells

**Figure 8:**
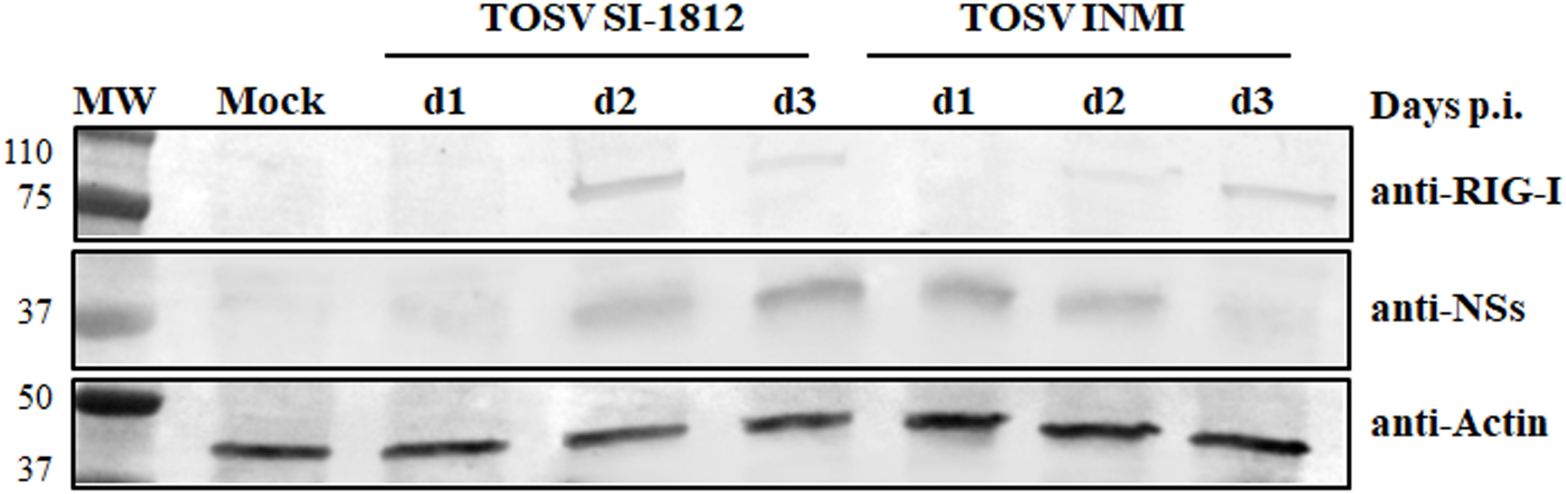
RIG-I modulation by the two TOSV strains. Endogenous RIG-I protein expression was analyzed by immunoblotting in DBTRG.05MG cells, either mock-infected or infected with TOSV SI-1812 or INMI strains, at various time points p.i. Simultaneously, the expression of the viral nonstructural NS protein was evaluated using a specific antibody. To ensure proper comparison between samples, antiactin monoclonal antibody was utilized as a loading control.

Previous data showed that the inhibitory effect of TOSV NSs on RIG-I was involved in the signaling cascade for type I IFN production, leading to degradation of RIG-I upon binding. In this study, we examined whether the interaction between RIG-I and NSs could have a functional consequence when DBTRG.05MG cells were infected with TOSV INMI or SI-1812 strain. For this purpose, we assessed the presence of RIG-I and NSs in these infected cells at 24, 48, and 72 h p.i. As shown in **Figure 8**, the RIG-I protein became detectable at 2 days p.i., while NSs were more prominent at 3 days p.i. in cells infected with the SI-1812 strain. In contrast, RIG-I was observed only at 3 days p.i., whereas NSs were strongly expressed as early as 1 day p.i. in cells infected with the INMI strain, suggesting that early and efficient expression of NSs in INMI-infected cells led to RIG-I degradation and hence impaired IFN-β response. This feature also confirmed that the different amount of IFN-β expressed by these cells depends on the TOSV strain used for infection.

## Discussion

It is well established that TOSV exhibits tropism for the CNS, causing aseptic acute meningitis, encephalitis, or meningoencephalitis (Charrel et al., 2012; Varani et al., 2015; Ayhan and Charrel, 2020). In this study, we used three human neural cell systems, i.e., DBTRG.05MG glioblastoma cells, ESC-derived cortical neurons, and brain organoids, to investigate *in vitro* the pathobiology and fitness of the TOSV SI-1812 and INMI strains (Cusi et al., 2005; Matusali et al., 2022). The TOSV INMI strain was isolated from the seminal fluid of a patient with an atypical clinical course, characterized by meningoencephalitis and persistent viral shedding in the seminal fluid for about 3 months. TOSV INMI infection efficiency, replication, CPE, and induction of the innate immune response were compared with the reference strain SI-1812 isolated from the CSF of a patient with acute meningoencephalitis. Both TOSV strains replicated and induced IFN-β production across all systems, but the INMI strain displayed slower growth kinetics (except in the glioblastoma cell line), leading to less CPE and less induction of IFN-β and other proinflammatory cytokines than the reference SI-1812 strain. In DBTRG.05MG cells, both TOSV strains exhibited similar replication kinetics over the first 4 days p.i., but INMI showed more pronounced growth by day 6 p.i. In ESC-derived neurons and hBOs, INMI displayed slower growth in the first 3 days but successively reached titers comparable to SI-1812. Notably, INMI persisted in organoids for up to 21 days, whereas infectious SI-1812 was no longer detectable in growth medium because of the massive disruption of the infected hBOs. This is in agreement with the enhanced capacity of the INMI strain to persist in the CNS *in vivo* (Matusali et al., 2022). Moreover, it is worth noting that SI-1812 infection induced the release of inflammatory factors such as IL-6 and IL-8 by glioblastoma cells, cortical neurons, and hBOs during injury, leading to brain damage (Cusi et al., 2016; Grebenciucova and VanHaerents, 2023).

Interestingly, INMI induced significantly lower levels of IFN-β across all time points and cell systems compared to SI-1812, with the most pronounced differences observed in hBOs. Our *in vitro* findings suggest potential implications for *in vivo* infection dynamics. The TOSV INMI strain was characterized by slower initial growth and elicited a weaker innate immune response. This attenuated immune activation might have facilitated the persistence of the virus within the human host. In contrast, the SI-1812 strain replicated faster, inducing a stronger immune response that limited its persistence in the host cells and its spreading in different body tissues. Based on these data, we also analyzed the expression of TOSV NS protein in infected cells. The role of TOSV NSs in counteracting innate immunity has been documented (Gori-Savellini et al., 2013; Gori Savellini et al., 2015; Gori Savellini et al., 2019), and the temporal kinetics of NSs expression should be considered. In cells infected with the SI-1812 strain, RIG-I expression was detectable at 24–48 h p.i., likely because NS accumulation became more evident only at 72 h p.i. (**Figure 8**). This suggests that NSs act as an antagonist mainly during the early phases of infection, after which Toll-like receptor 3 (TLR3) and other pattern recognition receptors contribute to IFN-β induction. Conversely, in cells infected with the INMI strain, NSs was expressed earlier, within 48h p.i., resulting in a delayed appearance of RIG-I, promoting its proteasomal degradation and, consequently, a delay in IFN-β expression at 72 h p.i. To identify the genetic basis of the different phenotypes of the two TOSV strains, we performed a whole-genome sequencing (GenBank Accession Nos. OR105998, MZ643217.1).

The NS proteins of the two virus strains differ by 9 amino acids distributed along the sequence. In the NS sequence of the INMI strain, a mutation (L_286_P) occurs within the TLQ motif (a.a. 285–287), which has previously been identified as critical for protein function and may significantly affect its activity (Gori Savellini et al., 2015). This change may result in the accumulation of NSs protein in the cytoplasm, which mediates RIG-I ubiquitination and degradation, thus suppressing early immune responses (Gori-Savellini et al., 2013; Gori Savellini et al., 2019). Further studies are ongoing to clarify the impact of these genetic differences. Finally, immunofluorescence analysis of brain organoids revealed that SI-1812 caused substantial cytopathic effects, reducing organoid size, whereas INMI induced apoptosis, as indicated by cleaved caspase-3 staining, due to the activation of interferon-stimulated genes (ISGs), such as TNF-related apoptosis-inducing Ligand (TRAIL) and Fas ligand (FasL), which also promote programmed cell death (Chawla-Sarkar et al., 2003). While differential IFN-β activation may account for the observed behavioral differences between the strains, studies are ongoing to further elucidate the role of their genetic differences.

## Data Availability

The raw data supporting the conclusions of this article will be made available by the authors, without undue reservation.
